# Formulation and evaluation of topical essential oil lotion as repellent against *Aedes aegypti*

**DOI:** 10.1016/j.crpvbd.2026.100354

**Published:** 2026-01-23

**Authors:** Nataya Sutthanont, Passanesh Sukphopetch, Rittipun Rungruang, Jiraporn Leanpolchareanchai, Raweewan Srisawat

**Affiliations:** a Department of Medical Entomology, Faculty of Tropical Medicine, Mahidol University, Bangkok, 10400, Thailand; b Department of Microbiology and Immunology, Faculty of Tropical Medicine, Mahidol University, Bangkok, 10400, Thailand; c Department of Cosmetic Science, Faculty of Science and Technology, Suan Dusit University, Bangkok, 10700, Thailand; d Department of Pharmacy, Faculty of Pharmacy, Mahidol University, Bangkok, 10400, Thailand

**Keywords:** *Aedes aegypti*, Mosquito repellent, Citronella, Patchouli, Sage, Plant-based formulation

## Abstract

Mosquito-borne diseases, particularly dengue transmitted by *Aedes aegypti*, remain a major public health challenge, underscoring the urgent need for safe and effective plant-based repellents. We developed and evaluated a new lotion containing 15% w/w of a blend of citronella, patchouli, and sage essential oils, focusing on both skin safety and repellent efficacy. Skin compatibility was confirmed in 16 volunteers *via* patch testing at 30 min, 48 h, and 96 h post-application, with no irritation or allergic reactions observed. Repellent activity was assessed using the WHO-recommended Arm-in-Cage (AIC) method. The lotion achieved a mean complete protection time (CPT) of 103.13 ± 5.46 min and maintained > 90% protection over 3 h, with repellency rates of 100.0%, 99.41%, 94.19%, and 91.01% at 0, 1, 2, and 3 h, respectively. Across all volunteers, the mean repellency was 96.17 ± 0.81%, while biting rates remained below 14% at 3 h. These findings indicate that the essential-oil blend provides safe, sustained, and effective protection, supporting its potential as a natural alternative to synthetic repellents for personal protection against *Ae. aegypti*.

## Highlights

• Essential oil-based lotion developed as a natural mosquito repellent.
• Human patch tests showed no irritation or allergic reactions.
• The lotion maintains > 90 % repellency against *Aedes aegypti* for 3 h.
• Mean repellency of > 95 % was achieved in Arm-in-Cage testing.
• The lotion presents a safe, natural alternative to conventional repellents.

## Graphical abstract

**Figure:**
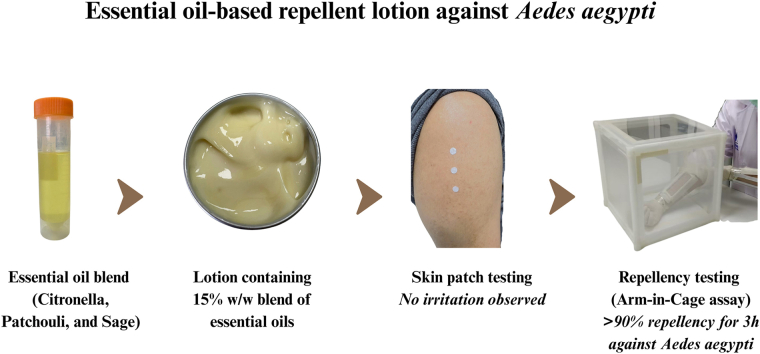

## 1 Introduction

Mosquitoes are globally distributed insects that not only cause nuisance through their bites but also serve as vectors for many life-threatening diseases (Benelli, 2015). Among these, *Aedes aegypti* is the principal transmitter of dengue hemorrhagic fever, spreading the virus through the bites of infected female mosquitoes (Briscoe, 1962; Pourzangiabadi et al., 2025). Dengue remains a major public health concern in tropical and subtropical regions, where the incidence has increased dramatically in recent decades. With no widely available antiviral treatment, preventive measures such as vector control and personal protection remain essential. In particular, personal protection through the use of mosquito repellents is a critical strategy for reducing human-vector contact and mitigating disease transmission risk (Lupi et al., 2013).

Synthetic repellents such as *N,N*-diethyl-meta-toluamide (DEET) have long been regarded as the gold standard for personal protection. However, concerns about potential toxicity, allergic reactions, and environmental persistence have raised interest in natural alternatives (Roy et al., 2017). Additionally, plant-derived repellents support sustainability, defined in this study as the use of renewable, biodegradable ingredients with lower ecological persistence (Jyotsna et al., 2024). Historically, humans have relied on plant-based solutions, such as burning aromatic plants, applying botanical extracts, or using strongly scented oils, to deter mosquitoes (Noguera-Gahona et al., 2024). Ethnobotanical studies document the traditional use of essential oils with repellent properties, many of which have since been commercialized for personal protection (Chellappandian et al., 2018; Teshome et al., 2025).

In the Asia-Pacific region, essential oils from citronella, lemongrass, eucalyptus, and kaffir lime have been widely studied and shown to provide effective protection against *Aedes aegypti* and other mosquito vectors (Phasomkusolsil and Soonwera, 2011; Sritabutra and Soonwera, 2013; Phukerd and Soonwera, 2014). Beyond repellency, essential oils offer multiple skin-care benefits, including antimicrobial, anti-inflammatory, antioxidant, and wound-healing properties (Swamy et al., 2016; van Beek and Joulain, 2017; Kamle et al., 2019; Mutinda et al., 2023). In recent years, natural repellents have gained popularity as safer, non-toxic alternatives to synthetic products. Essential oils have long been valued for their therapeutic benefits for skin health. Citronella oil, for example, is widely recognized for its mosquito-repelling activity but also exhibits antifungal, antibacterial, and anti-inflammatory effects (Trongtokit et al., 2005; Kim et al., 2007). Patchouli oil supports wound healing, skin regeneration, and hydration (Swamy et al., 2016; van Beek and Joulain, 2017), while sage oil provides antioxidant, anti-inflammatory, and antimicrobial benefits (Gali-Muhtasib and Affara, 2000; Sienkiewicz et al., 2015). These multifunctional properties make essential oils promising candidates for natural repellent formulations. The effectiveness of essential oil-based repellents depends not only on the choice of active ingredients but also on formulation characteristics, which influence protection duration, stability, and user acceptability (Lee, 2018). Lotions, in particular, offer advantages such as ease of application, even skin coverage, and the potential to reduce rapid evaporation of volatile compounds.

In response to the demand for safe, effective plant-based repellents, this study developed a new lotion containing a blend of citronella, patchouli, and sage essential oils. The formulation was guided by the hypothesis that combining multiple essential oils within a lotion base may prolong repellency while reducing the required concentration of each component. This hypothesis is supported by previous reports showing that blends of essential oils with differing volatility profiles can produce additive or synergistic repellent effects (Trongtokit et al., 2005; Nerio et al., 2010), even though individual-oil comparisons were not conducted within the present study. Repellent activity was evaluated using the Arm-in-Cage (AIC) method recommended by the World Health Organization (WHO) under controlled laboratory conditions, and the formulation underwent both safety (patch testing) and efficacy assessments following Thailand Food and Drug Administration (Thai FDA) laboratory testing standards to provide robust evidence for its potential as a natural alternative to synthetic repellents.

## 2 Materials and methods

### 2.1 Mosquito rearing conditions

The laboratory colony of pathogen-free *Ae. aegypti* (Bora Bora strain) was used in this study. The colony was reared and maintained in the insectary of the Department of Medical Entomology, Faculty of Tropical Medicine, Mahidol University, under controlled conditions: temperature of 25 ± 2 °C, relative humidity of 65 ± 10%, and a 12:12 h light/dark cycle. In each tray, 200 larvae were reared in 1000 ml of dechlorinated water, with fish food powder (Optimum, Perfect Companion Group Co., Ltd., Bangkok, Thailand), supplied daily as larval feeding. When the pupae appeared, they were taken out of the rearing tray every day, placed in a plastic cup with water, and placed in an adult cage (20 × 20 × 30 cm^3^) until emergence. Cotton wool soaked in a 5% sugar solution was used to feed the adult mosquitoes; the cotton wool was replaced once a week. For testing, 250 non-blood-fed female mosquitoes, 5–7 days-old, were isolated and sugar-fasted for 8–12 h (WHO, 2009).

### 2.2 Essential oil preparation

A blend of natural essential oils with previously reported mosquito-repellent activity was selected based on previous findings (Sutthanont et al., 2022) and ethnobotanical evidence. Citronella (*Cymbopogon nardus*), patchouli (*Pogostemon cablin*), and sage (*Salvia officinalis*) essential oils were obtained from Krungthepchemi Co., Ltd. (Bangkok, Thailand). Each oil was supplied with a certificate of analysis confirming its purity (> 95%). To provide standardized chemical identification, the CAS (Chemical Abstracts Service) numbers for all three oils are provided in Table 1. To the best of our knowledge, this specific combination of essential oils has not been previously reported in a mosquito repellent formulation. The essential oils used in this study are summarized in Table 1. The exact oil ratio is currently under optimization and is being considered for intellectual property protection. Future work will focus on improving formulation stability and extending the duration of protection.

**Table 1:** Essential oils used in the study.

Common name	Scientific name	Family	CAS Number	Plant part used
Citronella	*Cymbopogon nardus* Linn.	Poaceae	8000-29-1	Leaves and stems
Patchouli	*Pogostemon cablin* Benth.	Lamiaceae	8014-09-3	Leaves
Sage	*Salvia officinalis* Linn.	Lamiaceae	8022-56-8	Leaves

### 2.3 Lotion formulation

A topical lotion formulation containing a 15% w/w blend of citronella, patchouli, and sage oils was prepared using an oil-in-water (o/w) emulsion system. All formulation-grade chemicals were obtained from Chanjao Longevity Co., Ltd. and Krungthepchemi Co., Ltd. (Bangkok, Thailand). The water phase consisted of purified water, disodium ethylenediaminetetraacetate (EDTA), glycerin, carbomer, and titanium dioxide, while the oil phase contained dicaprylyl ether, dimethicone, Bis-PEG/PPG-16/16 PEG/PPG16/16 dimethicone, polysorbate 20, and the essential oil blend. The water phase was stirred at 500 rpm using an overhead stirrer (RW20, IKA, Staufen, Germany) until all solids were fully dissolved. The oil phase was mixed separately at the same speed until becoming homogenous. The oil phase was then poured into the water phase and homogenized at 4000 *rpm* for 10 min using an RS-HGM 15720 homogenizer (Rising Source and Supply, Pathum Thani, Thailand) to form the emulsion. The preservative system (caprylhydroxamic acid, phenoxyethanol, and methylpropanediol) and stabilizers (magnesium aluminium silicate) were subsequently added. The final pH was adjusted to 5.5–6.5 using 10% (w/v) sodium hydroxide solution. Further experiments were conducted at least 24 h after the o/w emulsion was prepared. All ingredients are listed in Table 2.

**Table 2:** Composition of the repellent lotion.

Part	Ingredient	Amount (%w/w)	Function
A (Water phase)	Purified water	45.10	Solvent
	Disodium EDTA	0.10	Chelating agent
	Glycerin	5.00	Humectant
	Carbomer	0.50	Thickening agent
	Titanium dioxide	0.10	Opacifying agent
B (Oil phase)	Dicaprylyl ether	2.00	Emollient
	Dimethicone	2.00	Emollient
	Bis-PEG/PPG-16/16 PEG/PPG16/16 dimethicone	4.00	Emulsifier
	Polysorbate 20	25.00	Solubilizer
	Essential oil blend	15.00	Active ingredient (repellent agent)
C	Caprylhydroxamic acid (and) phenoxyethanol (and) methylpropanediol	1.00	Preservative
	Magnesium aluminum silicate	0.20	Stabilizer
	10% w/v NaOH solution	q.s. to pH 5.5–6.5	pH adjusting agent

*Abbreviation*: q.s., quantum satis (sufficient quantity).

### 2.4 Human volunteers

Sixteen volunteers (eight males and eight females) were recruited for the study. All participants completed a survey confirming they had no known skin reactions to mosquito bites or to any ingredients in the test products. Their ages ranged from 20 to 50 years, and each provided written informed consent and received participant information prior to repellency testing. To reduce bias, participants were instructed to avoid fragrances, lotions, smoking, or other repellent products for at least 12 h before and during the testing period. During the experiments, they were also advised to keep their treated arms exposed and away from clothing or other surfaces to ensure accurate repellency assessment.

### 2.5 Skin patch testing

The skin irritation potential of the repellent lotion was assessed using a standard patch test following the International Contact Dermatitis Research Group (ICDRG) guidelines (Fregert, 1974; Bruze and Svedman, 2025). A total of 10 mg of the repellent lotion was applied to the external side of the upper arm using a 0.6-mm diameter circular filter paper (Finn Chamber, SmartPractice, Phoenix, AZ, USA). The site was occluded with a waterproof patch (3M Tegaderm) for 48 h. Skin reactions were evaluated by a board-certified dermatologist at 30 min, 48 h (immediately after patch removal), and 96 h post-application. The test sites were visually examined for signs of irritation, including redness, itching, pain, or vesicle formation. Reactions were scored from 1+ (mild) to 3+ (severe) according to the ICDRG criteria (Table 3). If any adverse reaction occurred at any observation point, the site was rinsed with water, the study physician was notified immediately, and appropriate medical care was provided. Participants with adverse reactions were excluded from further repellency testing.

**Table 3:** Scoring criteria for skin reactions according to the International Contact Dermatitis Research Group (ICDRG) guidelines.

Score	Reaction description
+++	Extreme positive reaction: spreading, bullous, or ulcerative lesions
++	Strong positive reaction: erythema, edema, papules, or vesicles
+	Weak positive reaction: non-vesicular erythema, infiltration, possibly papules
?	Doubtful reaction: faint or macular erythema only
IR	Irritant reaction: sharp-bordered erythema, glazing, or pustules not consistent with an allergic response
–	Negative reaction: no erythema, itching, pain, edema, or vesicle formation observed

### 2.6 Repellent activity test

**Fig. 1:**
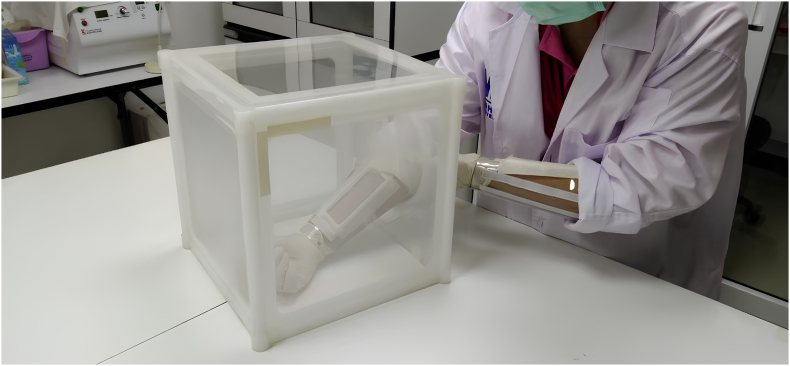
Arm-in-Cage (AIC) setup used for laboratory repellency testing. The AIC setup was used to evaluate the repellent lotion under controlled laboratory conditions. A volunteer's forearm was exposed to 250 female *Ae. aegypti* inside a 30 × 30 × 30 cm cage to assess mosquito landing and biting behavior under standardized test conditions.

The repellent lotion was evaluated under laboratory conditions using the human-bait technique. The procedure followed the Thai FDA testing standards (TISI, 2010) and Sutthanont et al. (2022), with selected elements adapted and modified from the WHO guidelines (WHO, 2009) to suit the evaluation of a topical lotion formulation. The study involved 16 healthy adult volunteers, tested in groups of four at random. Experiments were conducted under controlled conditions, with temperatures maintained at 25–27 °C and relative humidity of 60–80%. Before testing, volunteers washed their hands and arms with unscented soap, rinsed thoroughly with water, and then cleaned both arms with 70% alcohol. Each participant inserted one forearm into a mosquito cage (30 × 30 × 30 cm^3^) containing 250 female *Ae. aegypti* mosquitoes, aged 5–7 days. One forearm served as the control (untreated), while the other was treated with the test formulation. To standardize the test area, a 3 × 10 cm^2^ window was marked on the inner forearm (between the wrist and elbow), with both hands covered by plastic sleeves and protected by rubber gloves. The test began by exposing the untreated (control) left forearm to the mosquitoes. If at least 10 mosquito landings and/or probings occurred within 3 min, the repellency test proceeded. If fewer than 10 mosquitoes responded, a new cage of mosquitoes was used. For repellency testing, 100 mg of the formulation (3.3 mg/cm^2^) was applied evenly to the designated area of the treated right forearm and allowed to dry for 1 min. Both the control and treated arms were alternately inserted into the mosquito cage, with each arm exposed for 3 min to assess mosquito landing and biting behavior. The test was conducted at 30-min intervals for up to 3 h (Fig. 1). At each interval, the number of mosquitoes landing on or biting each arm was recorded. Repellency was assessed in accordance with the WHO (2009) and Thai FDA guidelines, using a 3-h AIC evaluation period. This duration provides sufficient mosquito biting pressure for reliable assessment, ensures volunteer safety and comfort, and facilitates comparability with other laboratory-based repellent studies (US EPA, 1999; Trongtokit et al., 2005; WHO, 2009).

### 2.7 Percentage repellency and biting

The percentage repellency represents the duration of repellent protection for each volunteer in terms of the percent reduction in probing landings attributed to the repellent. The formula was calculated as follows:

$$\%\;\text{Repellency}=\frac{\left(C\;‐\;T\right)}{C}\times 100$$

where C is the number of mosquitoes landing on the control arm, and T is the number of probing mosquitoes landing on the treated arm of the volunteer.

Conversely, the percentage biting (% biting) was calculated as:

$$\%\;\text{Biting}=\frac{T}{C}\times 100$$

This represents the proportion of mosquito landings that occurred on treated arms relative to untreated controls. Volunteers were exposed to mosquitoes every 30 min for 3 h.

The complete protection time (CPT) was defined as the duration until the second mosquito landed on or bit the treated arm. To determine the CPT of the mosquito repellent, the treated right arm of each volunteer was inserted into the test cage for 3 min. If no bites occurred, the arm was reinserted at 30-min intervals until the second mosquito probe was observed on the treated area. The time from the application of the test formulation to the second mosquito probe was recorded as the protection time.

### 2.8 Data analysis

Descriptive statistical analyses were used to summarize mosquito landing counts, percentage repellency, percentage biting, and CPT. For each outcome variable, the mean and standard error of the mean (SE) were calculated across all volunteers. As the objective of this study was to describe overall repellency performance rather than to compare demographic subgroups, all volunteer data were pooled for analysis. All calculations were performed using Microsoft Excel (Microsoft Corp., USA).

## 3 Results

### 3.1 Skin patch testing

Skin irritation and sensitization potential of the repellent lotion were evaluated in 16 healthy volunteers (8 males and 8 females) using the ICDRG patch test scoring system. All participants completed the 30-min, 48-h, or 96-h observation periods without any signs of allergic or irritant responses (Table 4). No erythema, pruritus, edema, vesicle formation, or bullae were observed at any of the evaluated time points. ICDRG scores remained at 0 for all participants throughout the study, indicating the absence of measurable skin reactions. None of the participants exhibited a response exceeding grade 1+ (mild), and no discontinuations occurred due to adverse events. The negative control site (vehicle-only base lotion) also showed no reactions in any subject. A full record of the individual participant results, including test codes and gender, is provided in Supplementary Table S1.

**Table 4:** Summary of allergy skin test results for the repellent lotion sample (*n* = 16 volunteers).

Time point	Observation outcome	ICDRG criteria
30 min	No visible reactions in all participants	–
48 h	No visible reactions in all participants	–
96 h	No visible reactions in all participants	–

*Note*: “–” indicates a negative reaction according to the ICDRG criteria.

### 3.2 Repellent activity

The repellent efficacy of the lotion was evaluated in 16 volunteers using the AIC method against *Ae. aegypti*. The primary measure of success, the CPT, averaged 103.13 ± 5.46 min, representing the interval from application until the second mosquito landing. Individual CPT results ranged from 60 to 150 min. Over the 3-h study period, the formulation demonstrated an overall repellency of 96.17%, indicating high protective efficacy against mosquito bites. When untreated control forearms were exposed to 250 mosquitoes for 3 min, the mean (± standard error of the mean, SE) number of landings was 155.75 ± 27.66 at 0 h, 167.63 ± 29.46 at 1 h, 162.13 ± 29.98 at 2 h, and 150.06 ± 24.97 at 3 h. In contrast, the treated arms maintained nearly complete protection.

Following CPT assessments, repellency tests were continued for up to 3 h. The percentage protection remained consistently high, with values of 100.00 ± 0.00% at 0 h, 99.41 ± 0.47% at 1 h, 94.19 ± 1.57% at 2 h, and 91.01 ± 1.58% at 3 h. Correspondingly, the percentage biting on treated arms remained very low, averaging 0.00 ± 0.00% at 0 h, 0.50 ± 0.38% at 1 h, 7.81 ± 1.85% at 2 h, and 13.75 ± 3.16% at 3 h. Table 5 summarizes these findings, highlighting the minimal biting rates and consistently high repellency across all observation periods. For transparency, detailed individual-level data for all volunteers are provided in Supplementary Table S2.

**Table 5:** Percentage of repellency and biting of repellent lotion over a 3-h period against *Ae. aegypti*.

Time post-application (min)	% Repellency (Mean ± SE)	% Biting (Mean ± SE)
0	100.00 ± 0.00	0.00 ± 0.00
60	99.41 ± 0.47	0.50 ± 0.38
120	94.19 ± 1.57	7.81 ± 1.85
180	91.01 ± 1.58	13.75 ± 3.16
Overall average	96.17 ± 0.81	3.83 ± 0.81

*Note*: Mean ± standard error of the mean (SE) calculated for 16 volunteers.

## 4 Discussion

The repellent lotion was rigorously evaluated for both safety and efficacy using standard patch testing and the AIC method against *Ae. aegypti*. In 16 volunteers, the skin patch test revealed no signs of allergic reactions at 30 min, 48 h, or 96 h post-application. None experienced redness, itching, pain, or vesicle formation, confirming the formulation's compatibility with human skin and its safety for topical use (Hussain Shah et al., 2013). Regarding protective performance, the lotion demonstrated strong and sustained repellency throughout the 3-h evaluation. CPT averaged 103.13 min, with individual values ranging from 60 to 150 min. Mean repellency remained at 100% immediately after application, decreasing only slightly to 99.41% at 1 h, 94.19% at 2 h, and 91.01% at 3 h, corresponding to minimal biting rates of 0%, 0.50%, 7.81%, and 13.75%, respectively. Continuous high mosquito biting pressure was confirmed by untreated control arms, which recorded 150–168 landings at the same intervals. Overall, the lotion maintained 96.17% repellency across all volunteers, clearly demonstrating its robust protective efficacy. The high repellency likely derives from the blend of citronella, patchouli, and sage essential oils. Each oil has documented repellent properties, for instance, pure patchouli oil (100%) affords complete protection for up to 2 h, though high volatility limits the persistence of most essential oils (Trongtokit et al., 2005). Our formulation maintained > 96% protection for 3 h using only 15% w/w essential oils, suggesting that formulation design may contribute to stabilizing volatile constituents and achieving high laboratory efficacy at a relatively low, skin-tolerable concentration (Harris, 2002; Caesar and Cech, 2019).

Although citronella-based repellents are widely available, their reported performance varies considerably depending on formulation type, essential oil concentration, volatility control, and the inclusion of fixatives or stabilizing excipients. Moreover, many commercial products do not disclose complete compositional details, and independently generated efficacy data obtained under standardized AIC conditions remain limited (Nerio et al., 2010; Maia and Moore, 2011). Previous studies have consistently shown that citronella-based formulations often exhibit a rapid decline in protection, particularly when used alone or at low concentrations, due to the high volatility of citronella oil (Tawatsin et al., 2001; Fradin and Day, 2002; Songkro et al., 2012). In contrast, the present formulation maintained > 96% repellency for 3 h at a comparatively low total essential oil concentration, yielding laboratory efficacy comparable to that reported for higher-dose essential-oil formulations in the literature.

Although this study was not designed as a direct head-to-head comparison with commercial products, these findings demonstrate that a carefully formulated essential-oil blend can provide strong baseline efficacy while minimizing total active content, supporting its relevance as a scientifically characterized plant-based alternative. Two complementary mechanisms are likely responsible for this enhanced performance. First, the emulsion-based lotion base creates a semi-occlusive film on the skin, which slows the evaporation of active compounds and modulates their release kinetics, thereby extending the duration of protection (Kongkaew et al., 2011). Secondly, the inherent low volatility and higher molecular weight of patchouli oil act as a natural fixative, gradually releasing the more volatile citronella components and sustaining their repellent activity over time (Trongtokit et al., 2005). The botanical origins of these oils further justify their selection, as plants from the families Poaceae and Lamiaceae are well recognized for strong repellent activity (Amer and Mehlhorn, 2006). Moreover, plant-derived repellents offer additional advantages in terms of sustainability. Essential oils are inherently biodegradable, exhibit low environmental persistence, and can be sourced from renewable agricultural crops, distinguishing them from some synthetic chemicals that may pose concerns related to toxicity and long-term ecological impact (Isman, 2006). Incorporating renewable, biodegradable ingredients, therefore, aligns with broader sustainability goals while supporting the development of safer alternatives for mosquito control (Regnault-Roger, 1997). In this formulation, citronella (*C. nardus*) represents Poaceae, while patchouli (*P. cablin*) and sage (*S. officinalis*) belong to Lamiaceae. Furthermore, formulation excipients likely contributed to the observed efficacy. Thickening agents such as carbomer slowed the evaporation of volatile oils and controlled their release (Barel et al., 2014; Sheskey et al., 2017), while emollients (dicaprylyl ether and dimethicone) ensured even skin coverage and improved release characteristics (Berkey et al., 2020; Franco-Gil et al., 2024). Glycerin, serving as a humectant, maintained moisture and formulation stability (Barel et al., 2014; Sheskey et al., 2017). Opacifiers such as titanium dioxide enhanced appearance and opacity and may have shielded essential oils from photodegradation (Popov et al., 2005). Stabilizers including disodium EDTA and magnesium aluminum silicate protected oils from degradation (Akhtar et al., 2010; Stoleru and Brebu, 2021), while surfactants, including polysorbate 20 as solubilizer and Bis-PEG/PPG-16/16 PEG/PPG16/16 dimethicone as an emulsifier, supported formulation homogeneity and helped maintain consistent repellency (Becher, 2001; Rhein et al., 2006). Finally, preservatives and pH adjustment maintained product stability and skin compatibility (Steinberg, 2006). Collectively, these elements yielded a safe, effective, and stable formulation, complementing the activity of the essential oils and prolonging repellency. Future studies should include larger and more diverse cohorts, field evaluations under real-world conditions, and testing against additional mosquito species to strengthen the evidence for broad-spectrum efficacy.

Although the lotion achieved a mean CPT of approximately 100 min and maintained > 90 % repellency, further optimization is required to extend its protective duration. This study did not include a direct comparison with commercial repellent products, as the primary objective was to establish the safety profile and baseline efficacy of the newly formulated essential-oil lotion. Future research will incorporate standardized comparisons with marketed repellents to better define relative performance and support product positioning. Overall, the lotion demonstrated good safety and reliable protection against *Ae. aegypti*, supporting its promise as a natural alternative to synthetic repellents and providing a strong foundation for future formulation refinement and clinical development.

## 5 Conclusions

This study developed and rigorously evaluated a new plant-based repellent lotion formulated with a blend of citronella, patchouli, and sage essential oils. The formulation proved safe for human topical use, with no evidence of skin irritation or allergic reactions observed during patch testing. Crucially, it provided consistent protection against *Ae. aegypti*, maintaining over 96% repellency throughout the 3-h laboratory evaluation, with a mean CPT of approximately 100 min. These findings demonstrate that combining multiple essential oils in a lotion base can deliver high repellency at a relatively low total concentration (15% w/w) while maintaining excellent skin tolerability. Overall, this plant-based formulation represents a potent, long-lasting, and safe natural alternative to synthetic repellents, offering a promising strategy for personal protection against mosquito bites and mosquito-borne diseases.

## Statement on the use of AI-assisted technologies

During the preparation of this work, the authors used Grammarly (https://www.grammarly.com/) and QuillBot (https://quillbot.com/) in order to correct grammatical errors and improve readability. After using these tools, the authors reviewed and edited the content as needed and take full responsibility for the content of the publication.

## Ethical approval

This study was conducted in accordance with the ethical guidelines of the Faculty of Tropical Medicine, Mahidol University, Bangkok, Thailand. Ethical approval for human testing using AIC method was obtained under Certificate of Ethical Approval MUTM 2024-091-01. Ethical approval for animal use was granted under Certificate No. FTM-ACUC 020/2024 by the Institutional Animal Care and Use Committee (FTM-ACUC) of the Faculty of Tropical Medicine, Mahidol University. All participants provided written informed consent and received participant information prior to repellency testing.

## CRediT authorship contribution statement

**Nataya Sutthanont:** Conceptualization, Data curation, Formal analysis, Funding acquisition, Investigation, Methodology, Project administration, Resources, Supervision, Validation, Visualization, Writing – original draft, Writing – review & editing. **Passanesh Sukphopetch:** Conceptualization, Funding acquisition, Methodology, Supervision, Validation, Writing – review & editing. **Rittipun Rungruang:** Formal analysis, Methodology, Visualization, Writing – review & editing. **Jiraporn Leanpolchareanchai:** Conceptualization, Data curation, Funding acquisition, Investigation, Methodology, Resources, Supervision, Validation, Writing – review & editing. **Raweewan Srisawat:** Conceptualization, Data curation, Funding acquisition, Investigation, Methodology, Resources, Supervision, Validation, Writing – review & editing.

## **Funding**

Financial support for this study was provided by the 10.13039/501100004156Mahidol University Fundamental Fund (fiscal year 2025), funded by the National Science Research and Innovation Fund (NSRF).

## Declaration of competing interests

The authors declare that they have no known competing financial interests or personal relationships that could have appeared to influence the work reported in this paper.

## Data Availability

The data supporting the conclusions of this article are included within the article and its supplementary files.
