# Supplementary material for: Formulation and evaluation of topical essential oil lotion as repellent against Aedes aegypti
Source: Curr Res Parasitol Vector Borne Dis. 2026 Jan 23;9:100354. doi: 10.1016/j.crpvbd.2026.100354 (PMC12925053; doi:10.1016/j.crpvbd.2026.100354)
Supplement: Multimedia component 1 [file mmc1.pdf]

Supplementary Information

Table S1. Detailed allergy skin test results for each volunteer

Volunteer code	Gender	Reaction to repellent lotion		
		**30 min**	**48 h**	**96 h**
V1	Female	–	–	–
V2	Male	–	–	–
V3	Female	–	–	–
V4	Male	–	–	–
V5	Female	–	–	–
V6	Female	–	–	–
V7	Male	–	–	–
V8	Male	–	–	–
V9	Female	–	–	–
V10	Male	–	–	–
V11	Female	–	–	–
V12	Male	–	–	–
V13	Female	–	–	–
V14	Male	–	–	–
V15	Female	–	–	–
V16	Male	–	–	–

– indicates a negative reaction according to the ICDRG criteria.

Table S2. Repellency and complete protection time of the repellent lotion against *Aedes aegypti*

Volunteers	Repellency (%) (±SE) at hours after treatment												Overall % Repellency	% Biting	CPT (min)
	0 h			1 h			2 h			3 h					
	Control (*N*)	Treated (*N*)	% Repellency	Control (*N*)	Treated (*N*)	% Repellency	Control (*N*)	Treated (*N*)	% Repellency	Control (*N*)	Treated (*N*)	% Repellency			
V1	90	0	100.00	129	0	100.00	70	3	95.71	68	4	94.12	97.14	2.86	90.00
V2	344	0	100.00	376	0	100.00	353	24	93.20	256	45	82.42	93.27	6.73	90.00
V3	151	0	100.00	84	0	100.00	81	3	96.30	77	6	92.21	94.99	5.01	90.00
V4	27	0	100.00	81	6	92.59	52	14	73.08	30	6	80.00	86.20	13.80	60.00
V5	22	0	100.00	60	1	98.33	42	3	92.86	49	3	93.88	96.78	3.22	90.00
V6	54	0	100.00	77	0	100.00	92	2	97.83	82	2	97.56	98.92	1.08	120.00
V7	220	0	100.00	285	1	99.65	358	22	93.85	341	17	95.01	97.43	2.57	90.00
V8	60	0	100.00	24	0	100.00	31	2	93.55	28	1	96.43	97.64	2.36	120.00
V9	57	0	100.00	80	0	100.00	144	0	100.00	147	5	96.60	99.03	0.97	150.00
V10	97	0	100.00	100	0	100.00	64	1	98.44	94	3	96.81	98.63	1.37	90.00
V11	245	0	100.00	231	0	100.00	371	11	97.04	288	28	90.28	97.62	2.38	120.00
V12	101	0	100.00	97	0	100.00	94	11	88.30	101	24	76.24	92.22	7.78	120.00
V13	252	0	100.00	223	0	100.00	246	7	97.15	188	17	90.96	97.18	2.82	90.00
V14	145	0	100.00	131	0	100.00	146	3	97.95	154	11	92.86	97.81	2.19	120.00
V15	348	0	100.00	375	0	100.00	182	6	96.70	241	19	92.12	97.34	2.66	90.00
V16	279	0	100.00	329	0	100.00	268	13	95.15	257	29	88.72	96.46	3.54	120.00
**Mean ± SE**	**155.75 ± 27.66**	**0 ± 0.00**	**100.00 ± 0.00**	**167.63 ± 29.46**	**0.50 ± 0.38**	**99.41 ± 0.47**	**162.13 ± 29.98**	**7.81 ± 1.85**	**94.19 ± 1.57**	**150.06 ± 24.97**	**13.75 ± 3.16**	**91.01 ± 1.58**	**96.17 ± 0.81**	**3.83 ± 0.81**	**103.13 ± 5.46**

Values represent the percentage repellency and biting calculated for each volunteer at hourly intervals over 3 h, based on mosquito landings recorded on treated versus untreated control arms. Overall percentage repellency and biting are averaged across the 3-h test period. The complete protection time (CPT) is defined as the interval from application until the second mosquito landing on the treated arm. Data are expressed as mean ± standard error (SE).
